# Kitchen Ranges and Stoves

**Published:** 1893-03-25

**Authors:** 


### PRACTICAL DEPARTMENTS.

### KITCHEN RANGES AND STOVES.

The subject of cooking by gas, giving illustrations of various apparatus, was treated, as our readers will remember, in our issues for January 7th to 28th consecutively.

We have recently paid a visit to the Building Exhibition now being held at the Agricultural Hall, Islington, where various new inventions of a practical nature are to be seen. There would appear to be more competition in the matter of kitchen ranges and cooking apparatus than in any other line.

The Wilson Engineering Company have some very good specimens of ranges, grills, &c., on exhibition, both for coal and gas.

There is no doubt that cookery by gas is becoming more universal every day, and will probably in the near future almost entirely supersede the old methods; but there is still some prejudice in favour of coal fires, and when this is the case there remains the all-important question—where to find those ranges which are at once the most economical and the most satisfactory in general working.

The Wilson Company have several large ranges admirably adapted for use in hospitals and institutions. It is claimed for these stoves that they save 75 per cent. of fuel, being also practically smoke consuming, and it being possible to burn all kinds of fuel, including kitchen refuse, a distinct advantage from a sanitary point of view. These ranges are fitted with movable bottom gratings, for the speedy reducing or enlarging of the fire according to need. The front part of the fire can in this way be raised several inches. The stoves require no building in, and can therefore be placed on any part of the floor, in the centre of the kitchen if desired, and can be put in position ready for use in a few hours.

Of gas stoves, ovens, and grills, all kinds can be supplied. An enormous oven is exhibited which is capable of roasting an ox whole. One objection advanced with regard to cooking by gas, that of the possible tainting of the food from the fumes, has been met by the company by the invention of a system of heating whereby the gas burners are not inside the oven at all, but the heating is entirely managed by flues outside. The drawback to this arrangement is that some time is necessary to raise the oven to the requisite pitch of heat for cooking purposes, but this objection may be considered to be counter-balanced by a greater degree of safety—all fear of accidental explosions being obviated—and when once heated the oven can be maintained at the desired temperature with the expenditure of a very small amount of fuel. It is calculated that a dinner for 15 or 20 persons can be supplied at a cost of only twopence for gas.

For institutional purposes it would certainly seem that gas will finally carry the day, both for cleanliness and economy. To those interested in such matters we would suggest a visit to the show-rooms of the Wilson Company, 227, High Holborn, where any or all of these stoves may be seen.

